# Dissolution of epoxy thermosets *via* mild alcoholysis: the mechanism and kinetics study†

**DOI:** 10.1039/c7ra12787a

**Published:** 2018-01-04

**Authors:** Xiao Kuang, Qian Shi, Yunying Zhou, Zeang Zhao, Tiejun Wang, H. Jerry Qi

**Affiliations:** The George W. Woodruff School of Mechanical Engineering, Georgia Institute of Technology Atlanta GA 30332 USA qih@me.gatech.edu; Renewable Bioproduct Institute, Georgia Institute of Technology Atlanta GA 30332 USA; State Key Lab for Strength and Vibration of Mechanical Structures, School of Aerospace Engineering, Xian Jiaotong University Xian 710049 China; Department of Architectural Engineering, North China Institute of Aerospace Engineering Langfang 065000 China; College of Engineering, Peking University Beijing 100871 P. R. China

## Abstract

Thermoset dissolution based on degradable bond or exchange reaction has been recently utilized to achieve thermosetting polymer dissolution and recycling. In this paper, an industrial grade epoxy thermoset was utilized as a model system to demonstrate the thermoset dissolution *via* solvent assisted transesterification (or alcoholysis) with high efficiency under mild conditions. The anhydride–cured epoxy thermoset was depolymerized by selective ester bond cleavage in 1,5,7-triazabicyclo[4,4,0]dec-5-ene (TBD)–alcohol solution below 180 °C at ordinary pressure in less than two hours. The epoxy dissolution proceeded in a surface erosion mode *via* transesterification that was coupled with catalyst–alcohol diffusion. Based on this observation, a surface layer model containing three layers, namely the gel layer, solid swollen layer and pure polymer layer was used to analyze the thermoset dissolution kinetics. The epoxy dissolution kinetics was derived from the surface layer model, which could be used to predict the dissolution rate during the diffusion-rate-controlled dissolution process well. The results show that alcohols with larger diffusivity and better solubility lead to a higher alcohol/catalyst concentration in the gel layer and promote faster erosion and dissolution of epoxy. This is the first work to show that it is possible to depolymerize industrial epoxy using the principle of dynamic bonds with fast dissolution rate at mild temperature under ordinary pressure.

## Abstract 2

An industrial grade epoxy thermoset was utilized as a model system to demonstrate the thermoset dissolution *via* solvent assisted transesterification with high efficiency under mild conditions.

## Graphical abstract

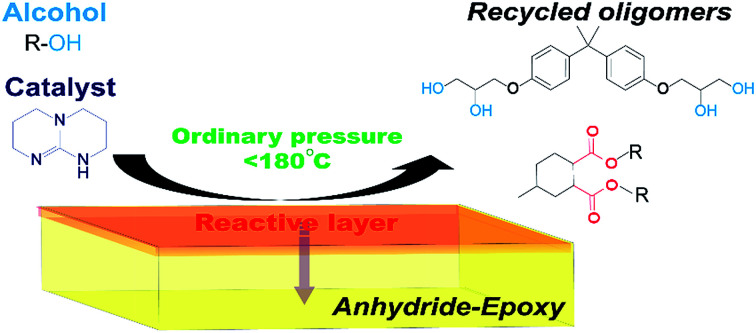

## 1. Introduction

Polymer dissolution in solvents is an important area of great interest in both polymer science and engineering due to its broad applications.^1^ Conventionally, chemically crosslinked polymers, known as thermosetting polymers, are difficult to dissolve except when using extremely strong chemicals at high temperature and high pressure.^2–5^ Once cured, the formed three-dimensional cross-linked network of a thermoset does not melt, making it difficult to degrade. The recent development of novel polymers with dynamic exchange reaction (ER) enables thermosets dissolution under mild conditions.^6–12^ The dynamic bonds activated by a specific stimulus or chemicals lead to network rearrangement and selective bond cleavage.^13–16^ These dynamic polymers have the desirable attributes of conventional thermosets, but also have more attractive attributes, such as self-healing and reprocessing of thermosets.^16–24^ Several dynamic reactions with different reaction mechanisms have been utilized for thermosets dissolution and recycling. Taynton *et al.* developed a dynamic polyimine system that can proceed transimination reaction with excess mount of diamine monomers and achieved full recycle of woven carbon fibers (CFs).^25,26^ Recently, our group developed a dissolution and recycling method by using transesterification reaction between hydroxyl group in ethylene glycol (EG) solvent and ester bond in the epoxy vitrimer.^27,28^ However, most of above dynamic thermosetting polymers are specialized polymers that need complex synthesis procedures or have compromised over-all properties at high temperature. For example, the epoxy vitrimer would lose its load carrying capability at high temperature due to ERs. Up to now, dissolving high performance thermosets by mild dissolution conditions is still a great challenge.^10^ It is also noted that the dissolution behavior and kinetics of thermosets dissolution are quite different from that of thermoplastics. First, the thermoset dissolution originates from the mass transportation of cleaved segments due to degradation reaction and the dissolution rate directly dependents on the reaction kinetics at the interface. Second, the solvent (and catalyst) diffusion into polymer determines the bond cleavage rate and finally affect the dissolution rate. These unique phenomena make the thermoset dissolution complicated. A deeper understanding of the thermosets dissolution mechanism and kinetics allows for the improvement and optimization of potential applications.

The dissolution of uncross-linked thermoplastic polymers in solvents has been extensively studied. The early work outlined the surface layer formation during polymer dissolution.^29^ Ouano *et al.* proposed the first model for thermoplastic dissolution.^30^ With Fickian diffusion into the polymer, the dissolution is controlled by both disentanglement of the polymer chains and the polymer-chain diffusion through a boundary layer adjacent to the polymer–solvent interface. Two distinct boundaries or interfaces, namely the liquid–gel interface and swollen layer-pure polymer interface characterized by a sharp change in the concentration of the solvent were observed.^30,31^ In erodible polymer matrix, there are two moving boundaries, the eroding polymer front and diffusion front, which make mathematical analysis of the dissolution kinetics complicated.^32^ Several phenomenological models were developed to study the kinematics of polymer dissolution and important parameters that affect the dissolution process, such as solvent diffusion coefficient, polymer molecular weight, solvent thermodynamic solubility and temperature were revealed.^29,30,33^

In this work, we proposed a novel method to dissolve the anhydride–epoxy thermoset *via* depolymerization, which is a widely used industry-grade high performance thermosetting polymer. An organic catalyst–alcohol solution was utilized to depolymerize anhydride–cured epoxy resin by rapid transesterification (or alcoholysis) between ester bond and alcohol. The dissolution molecular mechanism is studied by analyzing the chemical structures of decomposed oligomers. By considering the alcoholysis kinetics and diffusion behavior, a theoretical model was proposed to analyze the thermoset dissolution kinetics. The effects of alcohol types and temperature on epoxy dissolution were also systematically investigated. To our best knowledge, this work is the first to show that it is possible to dissolve industrial epoxy at mild temperature under ordinary pressure with high efficiency. This work also provides new insights into thermosetting polymer dissolution.

## 2. Experimental

### 2.1 Materials

Bisphenol A diglycidyl ether (DGEBA) epoxy oligomer (Epon resin 828), accelerator composed of 2,4,6-tris-dimethylaminomethyl phenol (Epikure Curing Agent 3253) were kindly provided by Hexion Inc (Pueblo, CO, USA). Hexahydro-4-methylphthalic anhydride (HP), ethylene glycol (EG), diethylene glycol (DG), propylene glycol (PG), 2-ethyl-hexanol (2EH), ethylene glycol monobutyl ether (EGMBE) and 1,5,7-triazabicyclo[4,4,0]dec-5-ene (TBD), zinc acetylacetonate (Zn(Ac)_2_) and triphenylphosphine (PPh_3_) were purchased from Sigma-Aldrich (St. Louis, MO, USA) and used as received. Silicone mold releasing agent was purchased from Stoner (Quarryvile, PA, UAS). Sodium hydroxide (NaOH) was obtained from Avantor Performance Materials (Phillipsburg, NJ, USA).

### 2.2 Preparation of epoxy resin

100 parts by weight of DGEBA, 89 parts by weight of HP and 0.375 part by weight of accelerator were mixed by manually stirring in a baker. After this, the mixture was placed in vacuum to degas for 10 min and then was poured into a mold coated with silicone releasing agent. The reactive mixture was first pre-cured at 100 °C for 2 h and then post-cured at 150 °C for another 2 h.

### 2.3 Characterizations

For quantitative swelling and dissolution tests, cubic epoxy resin samples with the side length of 4–5 mm were soaked in catalyst–alcohol solution in a glass bottle (with the polymer content of 5 wt% in the solution). The glass container was then sealed with aluminum foil and placed in a heating oven to the designated temperature. At different time intervals, the samples were taken out and weighed to monitor the residual mass as a function of heating time.

Uniaxial tension tests were used to evaluate the mechanical properties of epoxy resin. The samples were cut into stripes (∼30 mm × 10 mm × 2 mm). The tests were performed on a universal material testing machine (Model Insight 10, MTS Inc., Eden Prairie, MN, USA) at room temperature. The tensile rate was 1 mm min^−1^ for all the cases. Dynamic mechanical analysis (DMA) was conducted on a DMA tester (model Q800, TA Instruments, Inc, New Castle, DE, USA) using a tension clamp.

^1^H NMR and ^13^C NMR measurements were conducted on a Bruker Avance III 400 (Billerica, MA, USA) at room temperature using DMSO-d_6_ as a solvent. Fourier Transform Infrared Spectroscopy (FTIR) spectra were recorded on a Nicolet iS50 FTIR spectrometer (Thermo Scientific, Waltham, MA, USA) by averaging 32 scans of signal at a resolution of 2 cm^−1^ in attenuated total reflectance mode.

## 3. Results and discussion

### 3.1 The molecular mechanism of epoxy dissolution

**Scheme 1:**
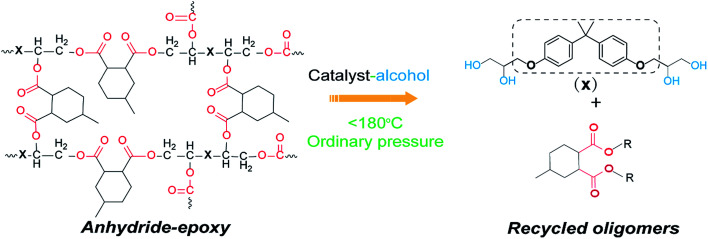
The proposed dissolution mechanism of anhydride–epoxy *via* alcoholysis in a catalyst–alcohol solution at mild conditions (ordinary pressure below 180 °C).

Anhydride–cured epoxy has been widely adopted in industry for fiber reinforced composites or electronic packaging.^34^ Our anhydride–cured epoxy resin shows a glass transition temperature (*T*_g_) of 160 °C and tensile strength of about 80 MPa (Fig. S1 and S2†). We use this high-performance epoxy thermoset as an example to show our dissolution approach. Scheme 1 describes the molecular mechanism of epoxy dissolution. The anhydride–cured epoxy containing ester bond is depolymerized into oligomers by a catalyst–alcohol system *via* transesterification that yields diester and tetra-alcohol.^35^

**Fig. 1:**
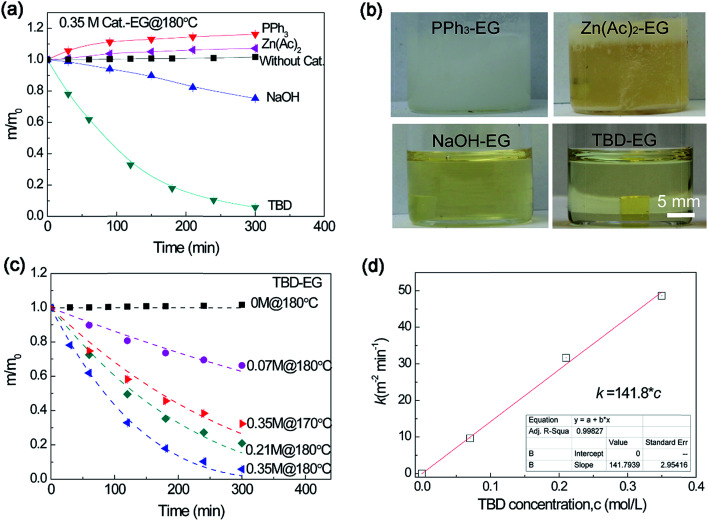
(a) Normalized residual mass of epoxy sample in catalyst–EG solutions using different catalysts. (b) The pictures showing the dissolution solution after immersing epoxy resin in different catalyst–EG solutions at 180 °C for 30 min. (c) Normalized residual mass of epoxy sample in 1,5,7-triazabicyclo[4,4,0]dec-5-ene (TBD)–ethylene glycol (EG) solution with different catalyst concentrations as a function of heating time at 180 °C (and 170 °C). (d) The dissolution rate constant derived from eqn (1)*versus* catalyst concentration.

The catalytic efficiency of several transesterification catalysts was tested for epoxy dissolution. Without any catalyst, the sample mass increases slightly after immersing in ethylene glycol (EG) at 180 °C for an extended time (more than 5 h) (Fig. 1a). The small increase in the mass is attributed to the diffusion of EG into the epoxy. In our previous works,^27,28^ Zn(Ac)_2_ was utilized as a catalyst in the epoxy vitrimer network, which has relatively low crosslinking density and low *T*_g_ (about 30 °C). For the highly crosslinked epoxy resin with high *T*_g_ in this work, it is extremely difficult to decompose the epoxy network using Zn(Ac)_2_ as a catalyst. The sample mass increases gradually in 0.35 M (mol L^−1^) Zn(Ac)_2_–EG systems at 180 °C for an extended time. A similar result was observed in another catalyst of PPh_3_. Although these transesterification catalysts can promote swelling to some extent, they show poor capability to cleave the ester bond for dissolution through transesterification reaction. For the strong inorganic base of NaOH, the sample mass decreases gradually with time. To expedite the dissolution process, we remedy the above the problem by using a strong organic base, 1,5,7-triazabicyclo[4,4,0]dec-5-ene (TBD) as a catalyst (with p*K*_a_ = 14.5). Fig. 1a also shows that TBD–EG solution enables much faster epoxy dissolution than that by NaOH. Comparing with other catalysts, TBD is well dissolved in EG, which is beneficial for alcoholysis reaction (Fig. 1b). Therefore, TBD can be used as a highly efficient catalyst for epoxy dissolution.

The effect of TBD catalyst concentration on epoxy dissolution was studied. As shown in Fig. 1c, the epoxy can be gradually decomposed in TBD–EG solution with different TBD concentrations at 180 °C and the dissolution rate increases with TBD concentrations. It was observed decreasing the temperature to 170 °C using the same catalyst concentration results in much slower dissolution. Thus the temperature plays an important role in the epoxy dissolution, which will be discussed later. To quantitatively study the dissolution rate, the epoxy dissolution kinetics was analyzed by the classical solid-state reaction theory (see ESI†). Using contracting cube model, the dissolution kinetics can be expressed as:^36^

1 1 − (1 − *α*)^1/3^ = 2*ka*^2^*t*

where *α* is the conversion fraction (the ratio of dissolved mass to original mass) at time *t*, *a* is the side length of cube (5 mm here), and *k* is the decomposition rate constant. The *k* value can be obtained by fitting experimental data using eqn (1). Interestingly, the *k* value shows a linear function of the catalyst concentration (Fig. 1d), *i.e.*, *k* = 141.8*c*, where *c* is the catalyst concentration. Since the chemical basis of epoxy dissolution is alcoholysis (the transesterification reaction between the alcohol and the ester bond), the dissolution rate is closely related to the transesterification rate. The transesterification rate constant *k*_ER_ usually increases with catalyst concentration.^37^ As a result, increasing catalyst concentration leads to an enhanced dissolution rate. The linear function between catalyst concentration and dissolution rate constant should be valid for different temperatures, but the coefficients may vary with temperatures.

**Fig. 2:**
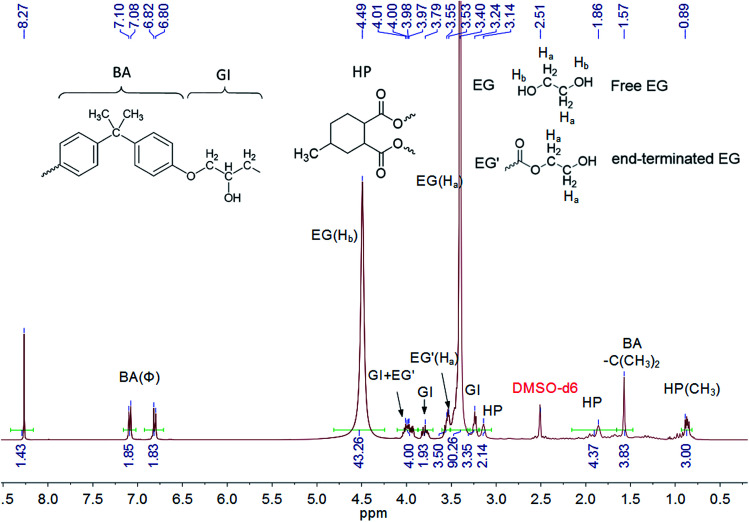
^1^H NMR spectrum of epoxy oligomer in 0.35 M TBD–EG solution with a solid content of 15 wt% during dissolution at 170 °C for 1 h.

To illustrate the catalytic mechanism of epoxy dissolution, the depolymerized product was analyzed. The chemical structure of the dissolution solution after thermal treatment was studied directly by NMR. The epoxy resin powder was added into 0.35 M TBD–EG solution with the solid content of 15 wt% and was fully dissolved at 170 °C within 1 h. Then the liquid NMR spectra was obtained by dissolving the above solution in DMSO-d_6_. As shown in Fig. 2, both free EG and end-terminated EG molecules are observed after treating the epoxy resin in TBD–EG solution. This means that the ester bond is cleaved by alcohol. This verifies the proposed alcoholysis reaction between alcohol and ester bond. The network is gradually depolymerized into soluble oligomers containing anhydride linkages (HP) and epoxy linkages (BA-GI). The degree of polymerization for the decomposed oligomer can be roughly evaluated *via* NMR by the proton ratios between end-terminated EG and anhydride linkage. Briefly, the peak at 3.53–3.55 ppm is attributed to the four protons from methylene moieties in two end-terminated EG and the peak at 0.89 ppm originates from the methyl moieties in HP linkages. Thus the average molar ratio between HP linkages and end-terminated EG is about 2.14 : 2 in the oligomer molecules, *i.e.* the average HP linkages in a recycled oligomer is 2.14 with two-terminated EG endings. The detailed peak assignments of proposed chemical structures and ^13^C NMR data further verified these results (Fig. S3†).

**Fig. 3:**
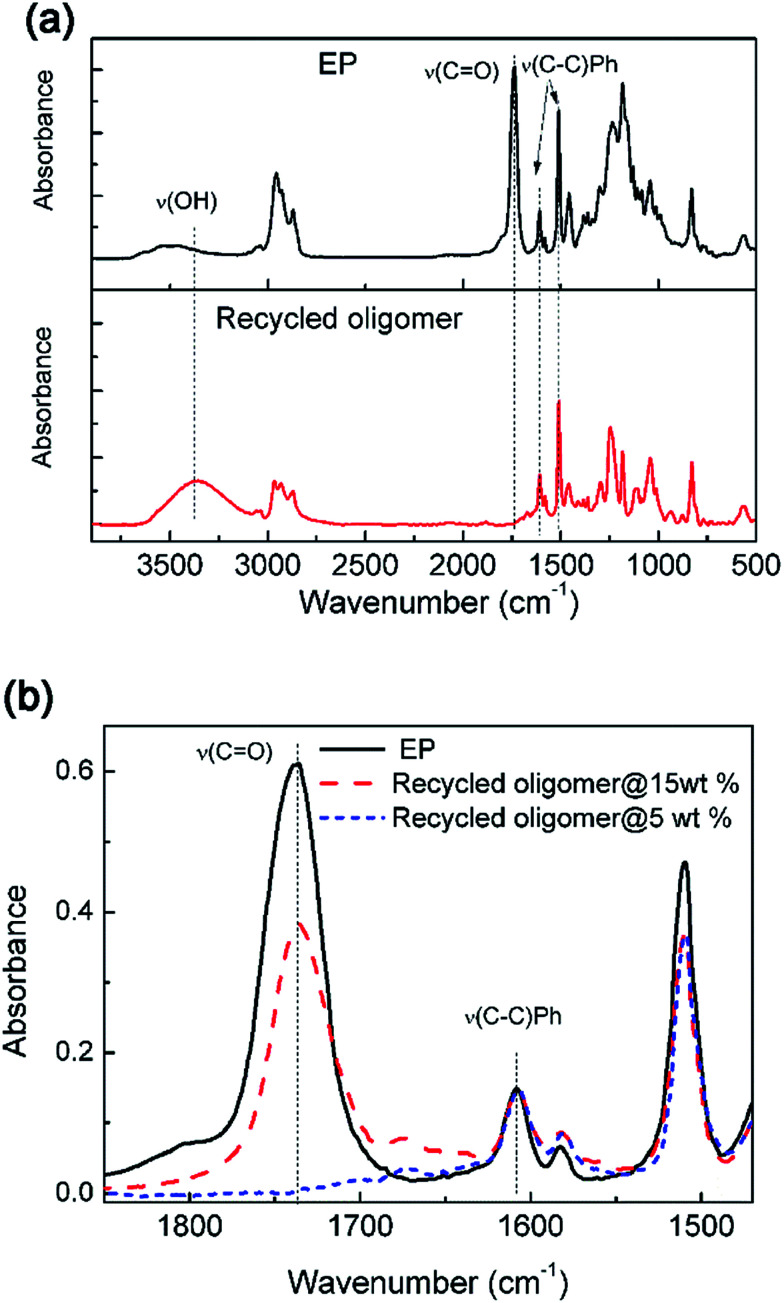
(a) The FTIR spectra of original epoxy resin and reclaimed oligomer after treating in 0.35 M TBD–EG solutions with solid content of 5 wt%. (b) The FTIR spectra showing the ester vibration (*v*_C=O_ = 1730 cm^−1^) of original and recycled oligomer after treating in 0.35 M TBD–EG solutions with solid content of 5 wt% and 15 wt%, respectively.

FTIR test was used to further analyze the chemical structure of recycled products. After dissolving different solid contents of epoxy resin in TBD–EG solution at 170 °C, the solutions were poured into water and the precipitates were recovered. The samples were dried and tested by FTIR. As shown in Fig. 3a, comparing with the fresh epoxy resin, the recycled oligomer with low solid content during dissolution shows obvious hydroxyl vibration (*v*_OH_). In addition, the characteristic peak of ester moieties (*v*_C=O_ = 1730 cm^−1^) disappeared in the recycled oligomers indicating complete elimination of ester moiety. This result indicates that the alcoholysis reaction is complete in case of low solid content (5 wt%) and all of the ester functions are cleaved and released. It is noted that the anhydride derivative diester molecules are water soluble and the DGEBA derivative tetra-alcohol is not water soluble. Consequently, the tetra-alcohol component is obtained in the organic phase after completing alcoholysis and the diester molecules stay in the water phase. Fig. 3b shows that the band of ester vibration (*v*_C=O_ = 1730 cm^−1^) is still obvious in the recycled oligomer using high solid content (15 wt%) during dissolution. Because the ester bond cleavage is incomplete for the recycled oligomer with high solid content and the limited time. Some HP linkages still exist in this recycled oligomer, which agrees with the NMR results.

**Scheme 2:**
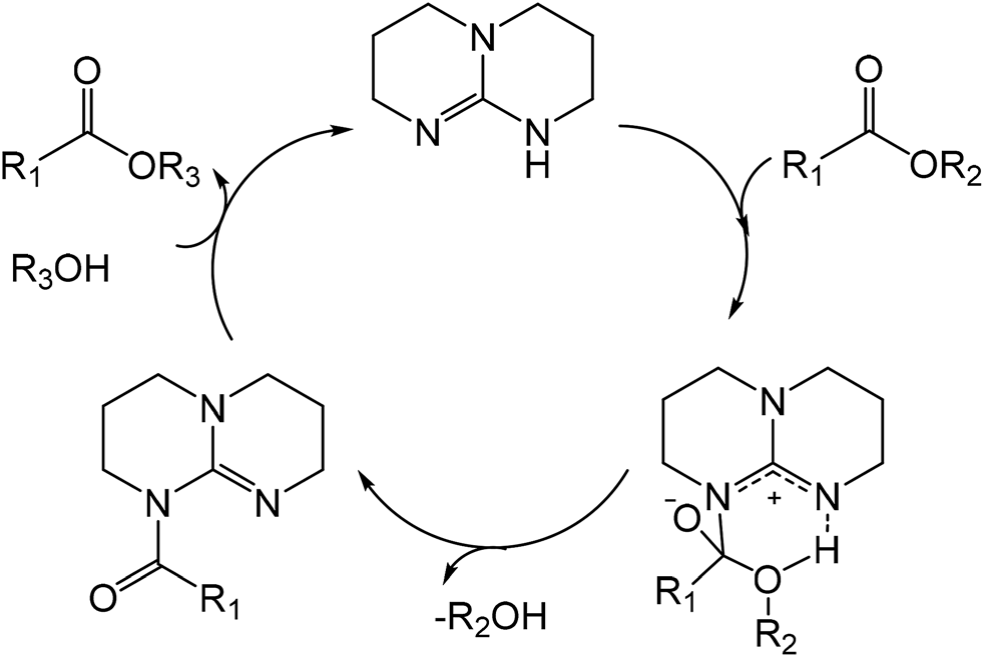
The proposed catalytic mechanism of alcoholysis reaction between ester and alcohol using TBD catalyst.

Based on the above results, we propose the catalytic mechanism of epoxy depolymerization in TBD–alcohol systems (Scheme 2). TBD first reacts with ester *via* nucleophilic attack of both disubstituted nitrogens at the carbonyl groups forming betaine-like intermediate.^38^ Then the proton transfer on the protonated nitrogen leads to the intermediate product of TBD amide and liberating the alcohol. With the incorporation of another alcohol, new ester can be rapidly formed by regenerating TBD.^39^ With the diffusion of TBD–alcohol into epoxy network, the alcohol would be continuously catalyzed to cleave the ester linkages in epoxy network, leading to rapid depolymerization of epoxy resin. Thus, the epoxy dissolution is a complex process where transesterification reaction is coupled with TBD–alcohol diffusion.

### 3.2 The surface layer model of epoxy dissolution

**Fig. 4:**
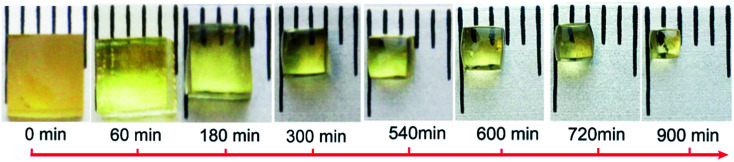
The appearance and size evolution of cubic epoxy samples after being soaked in 0.35 M TBD–EG for different times at 170 °C.

Based on the above discussed dissolution molecular mechanism, the dissolution kinetics was investigated. Fig. 4 shows the epoxy dissolution behavior in TBD–EG solution at 170 °C. It can be seen that the side length decreases but the cubic shape of the sample is maintained during dissolution. This indicates that the dissolution proceeds in a surface erosions mode. This is reasonable because EG is a poor solvent for epoxy as indicated by the large Flory–Hussein parameter (Table 1). The epoxy depolymerization reaction requires the diffusion of alcohol–catalyst. Thus the epoxy dissolution behavior is effected by both the transesterification reaction and the diffusion of polymer chains-solvent. In general, a thermoset dissolution process can be either reaction-rate-controlled, if the polymer–solvent diffusion rate in the interfacial layer is faster than the reaction rate, or diffusion-controlled, if the diffusion rate is slower than the reaction rate. Apparently, when using the highly efficient organic catalyst-poor solvent system, the dissolution would be a diffusion-rate-controlled process.

**Table 1:** The Hansen solubility parameters and physical properties of epoxy and several alcohols.^40,41^

Chemicals	[OH] (mol L^−1^)	*δ*_D_ a	*δ*_P_ a	*δ*_H_ a	*δ*_T_ a	*T*_b_ (°C)	*V*_1_ (cm^3^ mol^−1^)	*R*_a_/*R*_o_^b^	χ^c^
EG	35.8	17.0	11.0	26.0	33.0	196	55.8	1.15	0.58
PG	27.2	16.8	9.4	23.3	30.2	187	73.6	0.97	0.54
DG	21.1	16.6	12	20.7	29.1	245	94.9	0.82	0.50
2EH	6.4	15.9	3.3	11.8	20.1	183	156.6	0.57	0.41
EGMBE	7.6	16	5.1	12.3	20.8	171	131.6	0.49	0.24
Epoxy	—	18.4	9.4	10.1	23	—	—	—	—

a The unit is MPa^0.5^.

b *R*_o_ = 14.04.

c Predicted Flory–Huggins parameter between solvent and epoxy at 180 °C.

**Fig. 5:**
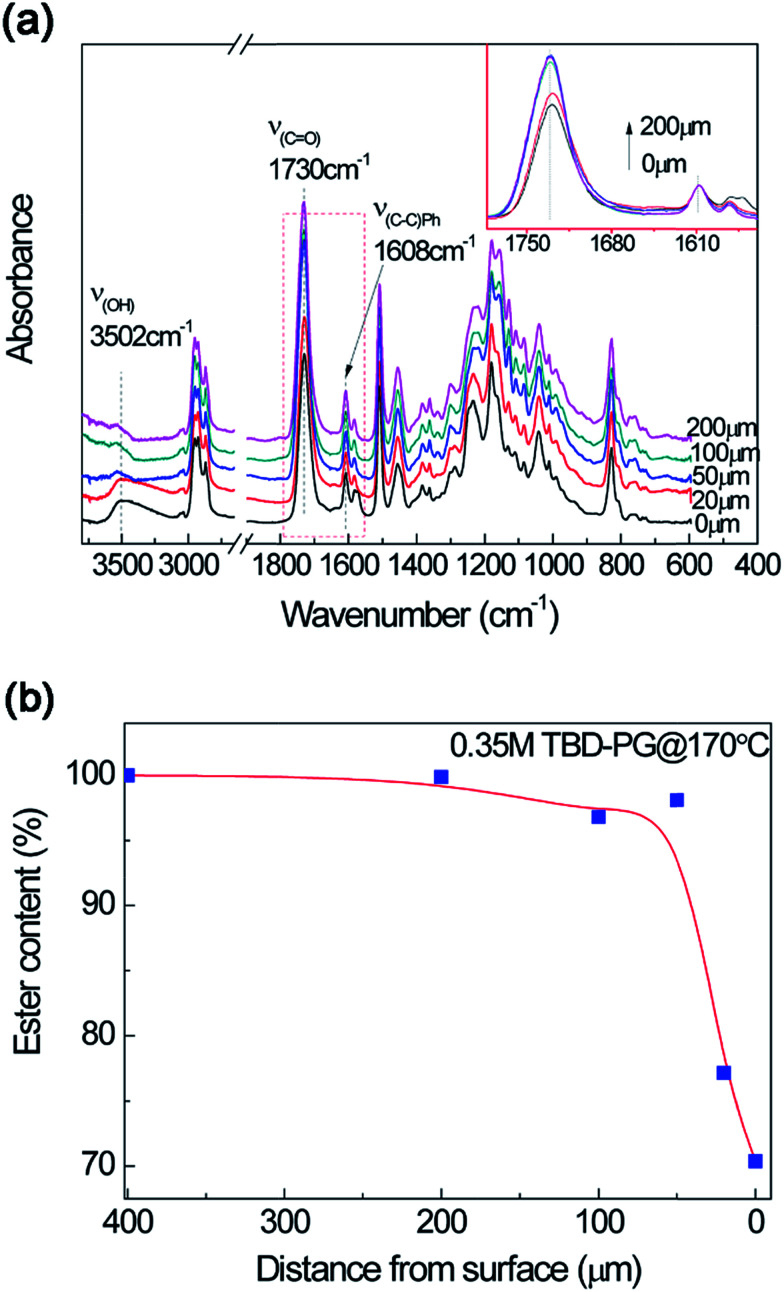
(a) FTIR spectra of epoxy in TBD–PG at different thickness after treating at 170 °C for 40 min. The inserted figure show the distance-dependent intensity of ester band. (b) Normalized ester content at different thickness after treating in TBD–PG at 170 °C for 40 min using ester band (*v*_C=O_ = 1730 cm^−1^) with phenol band (*v*_C=O_ = 1608 cm^−1^) as a reference by FTIR spectra.

The macromolecular structural evolution of the diffusional dissolution was followed by the FTIR test. After immersing the epoxy resin in TBD–alcohol solution at 170 °C for 40 min, the epoxy resin from different distance to the fresh surface was cut and tested. To obtain better results, the propylene glycol (PG) were utilized because of its relatively better solubility than that of EG. The effect of alcohol on epoxy dissolution will be discussed later. As shown in Fig. 5a, the band intensity of hydroxyl group is much stronger after treating in TBD–PG solution. Using the characteristic band of phenyl (*v*_C–C(Ph)_ = 1608 cm^−1^) as a reference, the relatively intensity of ester bond (*v*_C=O_ = 1730 cm^−1^) was obtained. Furthermore, the content of ester bond at different layers can be normalized by the ester bond content in bulk material and the distance-dependent ester bond content can be evaluated. Fig. 5b shows that the ester bond at the most outside surface layer is about 70% of the bulk. According to the classical gel theory,^42–44^ when the percentage of ester linkages drops lower than the gel point conversion, the oligomer or cluster gains sufficient mobility and diffuses into solvent. In a short time period, ester linkages in a very thin layer would effectively be cleaved and turned into segments with end-capped alcohol which can be finally be dissolved. These results indicate that the depolymerized oligomers with ester content lower than 70% may be dissolved into the solution. Fig. 5b also shows the ester content increases sharply within 50 μm, which seems to be a dissolution boundary. This is similar to the thermoplastics dissolution with a liquid-gel layer boundary showing highly nonlinear concentration dependence of the diffusion coefficient.^30,32,33^ The ester content increases slowly over 50 μm. With the distance larger than 200 μm from surface, the epoxy structures are comparable to the bulk polymer (not shown here).

**Fig. 6:**
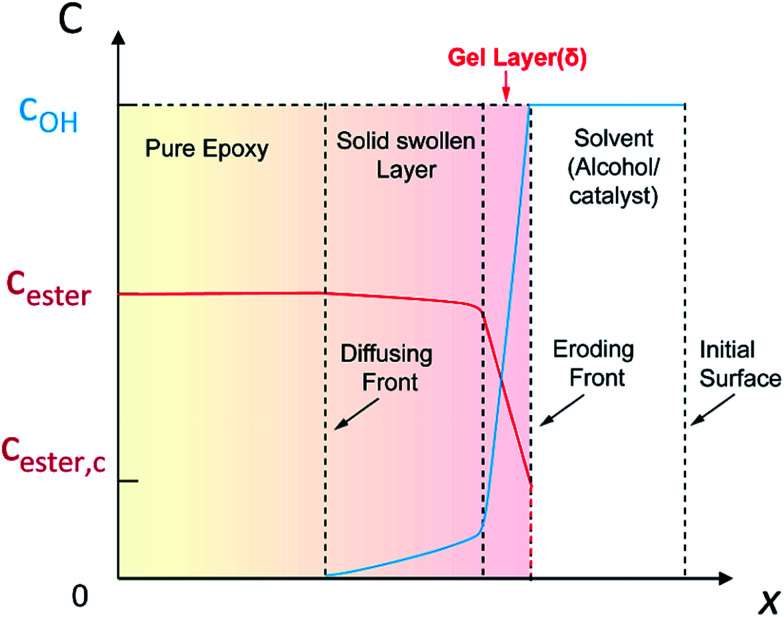
A schematic profile for the diffusional dissolution of epoxy thermosets *via* alcoholysis. The liquid–solid interface was divided into three layers: the gel layer, the solid swollen layer, and pure polymer layer. The gel layer with high solvent/catalyst concentration is highly reactive and dynamic, which is defined as the reactive layer. The layer thicknesses are schematic and are not to the scale.

In the thermosets dissolution, the diffusion induced alcoholysis and alcoholysis assists diffusion make it complex for mathematical analysis. Based on the above results, we propose a modified surface layer model to analyze epoxy dissolution in TBD–alcohol systems. The liquid–solid interface can be divided into three layers: the gel layer, the swollen layer, and the pure polymer layer (Fig. 6). The solvent would constantly diffuse into polymer and a diffusing front exists. With the bond cleavage and epoxy dissolution, the initial surface of polymer disappears and a new eroding front is formed. The gel layer contains the swollen polymer in a rubber-like state, in which the solvent/catalyst is relatively high. The swollen layer has a low alcohol concentration. The layer beyond swollen layer is the pure polymer. For the epoxy dissolution studied here, diffusion of TBD–alcohol into the anhydride–epoxy network activates the transesterification reaction. In addition, the reaction rate constant is linearly related to the catalyst concentration. According to the transesterification reaction kinetics (Scheme S2†), the reaction rate is proportional to the hydroxyl group concentration, the ester bond concentration, and the alcoholysis rate constant (*k*_ER_). As alcohols are relatively poor solvents for highly crosslinked epoxy with high glass transition, the swelling ratio is very low. For instance, the swelling ratio is as low as 1.06 in EG for 4 h at 180 °C. In the swollen layer, the EG concentration decreases to less than 6%. Beyond the gel layer, alcoholysis rate is so sluggish that almost no transesterification reaction occurs, due to low concentration of both alcohol and catalyst.

From this surface layer model, we propose the alcoholysis mainly occurs in the gel layer. During a certain time period, a dissolution equilibrium would be achieved and the concentration profile would be stable. For example, there is a critical ester bond concentration (*C*_ester_,_c_), which is the minimum value to maintain a cross-linked network. The maximum ester bond concentration is the bulk polymer. The moving distance of eroding front is equal to the thickness of the gel layer.^33^ As a result, the decomposition of epoxy would continuously proceed in the very thin gel layer (*δ*) that is defined as the reactive layer. It is hypothesized that dissolution fraction (*α*) is roughly proportional to the content of ester bonds cleaved by alcohol. Thus the dissolution kinetics can be correlated with the alcoholysis kinetics (see ESI†). Finally, the modified dissolution kinetics can be expressed as follows:2
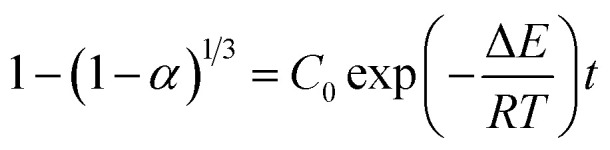
with 
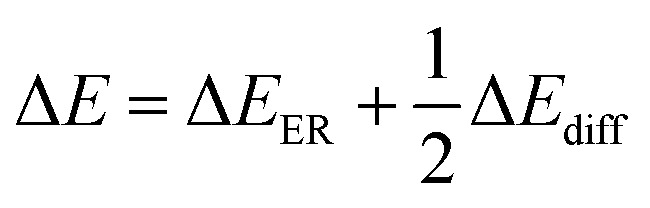
 and 
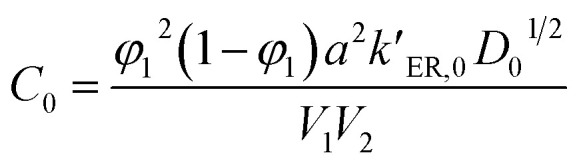
, where *C*_0_ is a constant, Δ*E*_ER_ is the transesterification reaction activation energy, Δ*E*_diff_ is the activation energy of alcohol diffusion into epoxy, Δ*E* is the apparent dissolution activation energy, *a* is the side length of cubic samples, *k*_ER,0_ is the ER rate constant at a reference temperature, *φ*_1_ is the solvent volume fraction in polymer, *D*_0_ is the diffusion coefficient at reference temperature, *V*_2_ is molar volume of polymer and *V*_1_ is molar volume of alcohol.

With the combination of the solid-state reaction kinetics (eqn (1)) and the proposed surface layer model (eqn (2)), we obtain the following equations:3
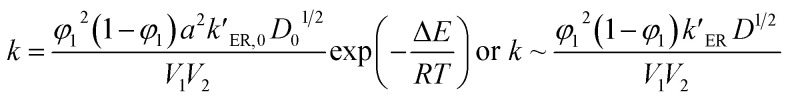
where *D* is mutual-diffusion coefficient of solvent–epoxy systems. The above equations are based on the relationship that the reactive later thickness (*δ*) is proportional to square root of diffusivity (*D*) for a given time step by Fick's second law of diffusion:^33^

4 *δ* ∼ *D*^1/2^

Form eqn (3), we find that the dissolution rate constant *k* is dependent on ER rate constant (*k*_ER_), the diffusion coefficient (*D*), alcohol concentration (1/*V*_1_) and the swelling ratios for the epoxy thermosets.

### 3.3 The effect of alcohols on epoxy dissolution

**Fig. 7:**
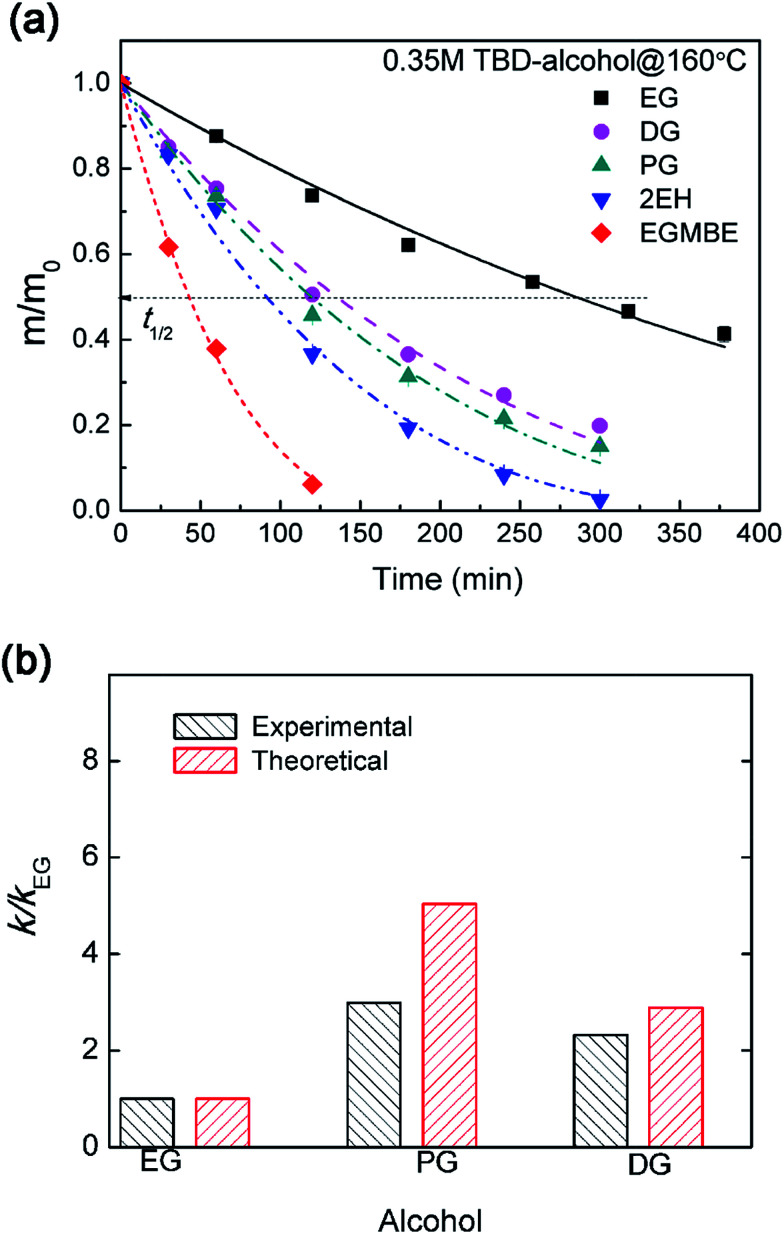
(a) Normalized residual mass of epoxy sample in 0.35 M TBD–alcohol solutions at 160 °C with different alcohols as a function of heating time. (b) The experimental and theoretical relative dissolution rate constant (*k*/*k*_EG_) for epoxy resin in 0.35 M TBD–alcohol systems with different alcohols.

The influence of alcohol types on epoxy dissolution was investigated. After incorporating TBD catalyst into alcohol, the epoxy resin can gradually be decomposed into soluble segments. With the mass transportation of soluble segments from the gel layer into alcohol, the epoxy resin can be gradually dissolved as indicated by the decreasing mass. As shown in Fig. 7a, EG and EGMBE show the slowest and the fastest epoxy dissolution, respectively. The dissolution rate can be quantitatively evaluated using the dissolution half time (*t*_1/2_) (Fig. S4†). The *t*_1/2_ is about 289 min for EG at 160 °C. The *t*_1/2_ decreases to as low as 42 min for TBD–EGMBE system. Similar trend was observed at lower temperature of 150 °C (Fig. S5†). It is noted that the time need to achieve swelling equilibrium in alcohol (Fig. S6†) is much longer than that for epoxy dissolution in TBD–alcohol solution. This result further suggests that the dissolution of highly cross-linked epoxy thermoset with high glass transition temperature in TBD–alcohol solution is a diffusion-rate-controlled process.

The effect of alcohol on the diffusion-rate-controlled dissolution was semi-quantitatively analyzed by the above surface layer model. As the *k*_ER_ is dependent on temperature and catalyst concentration, at the same temperature and catalyst concentration, eqn (3) can be simplified with *k* briefly expressed as follows:5
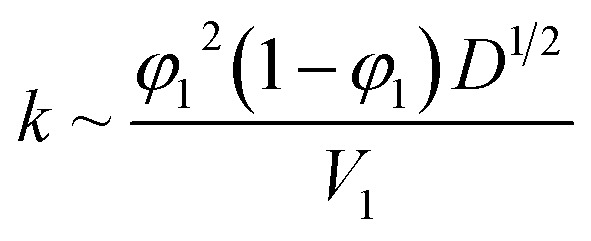


Obviously, the epoxy dissolution rate can be affected in terms of diffusivity and solubility as well as the hydroxyl concentration for different alcohol. The diffusivity of alcohols in epoxy was evaluated by swelling tests. The time dependent swelling ratio evolution of epoxy in different alcohols (without catalyst) reflects the alcohol diffusion kinetics. Fitting by Fick's second law, *D* value for different alcohol in epoxy was obtained. At 180 °C (higher than *T*_g_), 2EH shows higher *D* (3.5 × 10^−5^ mm^2^ s^−1^) than other alcohols (Fig. S6†). The continuous diffusion of solvent/catalyst into the gel layer of epoxy, alcoholysis proceeds to cleave the network. The cleavage rate of ester linkage in epoxy is related to the volume of reactive layer (or gel layer) (eqn S12†). Thus the equilibrium welling ratios, reflecting the solubility of epoxy in alcohol, play an important role in the dissolution rate. For example, a higher equilibrium welling ratio of 2EH (1.3) enables much fast epoxy dissolution than EG with lower value of 1.06 (Fig. S6†). The solubility of epoxy in alcohol can be explained using the three dimensional (3D) Hansen solubility parameters. 2EH is in the solubility sphere locating near the center suggesting good solubility (Fig. S7†). In contrast, EG is outside of epoxy solubility sphere indicating poor affinity to epoxy.

The above analysis as expressed by eqn (5) was validated by comparing the experimental and predicted value. As shown in Fig. 7b, the experimental and theoretical relative dissolution rate constant (*k*/*k*_EG_, using EG as a reference) for epoxy resin in 0.35 M TBD–alcohol systems have the same trend. Alcohols with larger swelling ratios and diffusivity lead to higher alcohol–catalyst concentration in the gel layer, which facilitates faster erosion of the gel layer. It should be noted that this surface layer model can predict the dissolution behavior well for relatively poor solvent or diffusion-rate-controlled process. For good solvent, however, the coupling between diffusion and dissolution occur in a larger thickness with obvious swelling. Thus the dissolution process would gradually shift to reaction-rate-controlled process. In the case of good solvent, more sophisticated mathematical analysis by considering both of swelling and diffusion–reaction coupling is need to analyze the dissolution kinetics.

### 3.4 The effect of temperature on epoxy dissolution

**Fig. 8:**
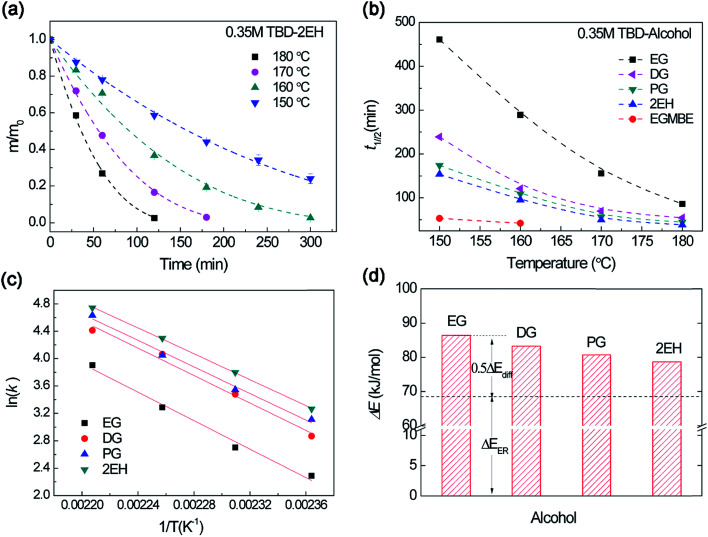
(a) Normalized residual mass of epoxy sample in 0.35 M TBD–2EH solution at different temperature as a function of heating time. Dash lines are the fitting curves by eqn (1). (b) The dissolution half time (*t*_1/2_) *versus* temperature for different alcohols. (c) Arrhenius plot of dissolution rate constant in TBD–alcohol solutions. (d) The dissolution activation energy for different TBD–alcohols solutions derived from Arrhenius law. The base line is the transesterification activation energy of about 68 kJ mol^−1^.

We further investigated the influence of temperature on the epoxy dissolution. As shown in Fig. 8a, at elevated temperatures, ER was activated in the TBD–2EH and the mass gradually decreased with decomposition process. Higher temperature affords faster dissolution for different alcohols (Fig. S8†). The *t*_1/2_ of different alcohols was plotted with temperature (Fig. 8b). The *t*_1/2_ decreases with increasing temperature for all alcohol. However, the *t*_1/2_ shows different temperature sensitivity. To quantitatively evaluate the temperature dependent dissolution behavior, the dissolution rate constants at different temperatures were derived by fitting with eqn (1). Fig. 8c depicts the temperature dependent dissolution rate constant fitted by the Arrhenius law. According to eqn (2), the decomposition activation energy (Δ*E*) for epoxy dissolution in TBD–alcohols was evaluated to be 78.7–86.4 kJ mol^−1^. This value is larger than the transesterification activation energy (Δ*E*_a_ ∼ 68 kJ mol^−1^).^45,46^ Because both the ER activation energy (Δ*E*_ER_) and diffusion activation energy (Δ*E*_diff_) contribute to the dissolution activation energy (eqn (2)). Using the same Δ*E*_ER_ as a baseline, the Δ*E*_diff_ can be roughly evaluated (Fig. 8d). The alcohol with better solubility show lower Δ*E*_diff_. For example, the EG show an Δ*E*_diff_ about 36.8 kJ mol^−1^, which are in the same range from literature.^47^ Therefore both diffusion and ER rate are affected by temperature during epoxy dissolution.

## 4. Conclusion

In summary, the anhydride–epoxy thermoset was dissolved *via* solvent assisted dynamic transesterification and a surface layer model was proposed to reveal the dissolution kinetics. TBD–alcohol solution is highly efficient to cleave the ester bond in anhydride–cured epoxy thermosets, leading to epoxy dissolution below 180 °C at ordinary pressure. The thermoset dissolution enabled by transesterification is coupled with catalyst–alcohol diffusion. A surface layer structure containing the gel layer, solid swollen layer and pure polymer layer was proposed. The epoxy dissolution occurs in the gel layer (or reactive layer) as indicated by a moving eroding front. For diffusion-rate-controlled dissolution in TBD/alcohol systems, the dissolution rate can be well predicted by the surface layer model. Alcohols with better solubility to epoxy has a larger solvent molecules diffusion and show higher alcohol–catalyst concentration in the gel layer promoting faster dissolution. This approach can be applied to other thermosetting polymers containing ester linkages, such as polyester. The on-going work is to further enhance the diffusion and swelling process toward even faster thermosets dissolution. The methodology developed in this work provides insight in solvent assisted thermosets dissolution or depolymerization *via* dynamic reaction.

## Conflicts of interest

The authors declare no competing financial interests.

## Supplementary Material

RA-008-C7RA12787A-s001

## References

[cit1] Miller-Chou B. A., Koenig J. L. (2003). Prog. Polym. Sci..

[cit2] Morales Ibarra R., Sasaki M., Goto M., Quitain A. T., García Montes S. M., Aguilar-Garib J. A. (2015). J. Mater. Cycles Waste Manage..

[cit3] Piñero-Hernanz R., García-Serna J., Dodds C., Hyde J., Poliakoff M., Cocero M. J., Kingman S., Pickering S., Lester E. (2008). J. Supercrit. Fluids.

[cit4] Yan H., Lu C.-X., Jing D.-Q., Chang C.-B., Liu N.-X., Hou X.-L. (2016). New Carbon Mater..

[cit5] Henry L., Schneller A., Doerfler J., Mueller W. M., Aymonier C., Horn S. (2016). Polym. Degrad. Stab..

[cit6] Hashimoto T., Meiji H., Urushisaki M., Sakaguchi T., Kawabe K., Tsuchida C., Kondo K. (2012). J. Polym. Sci., Part A: Polym. Chem..

[cit7] Wang Y., Cui X., Yang Q., Deng T., Wang Y., Yang Y., Jia S., Qin Z., Hou X. (2015). Green Chem..

[cit8] Ma S., Webster D. C., Jabeen F. (2016). Macromolecules.

[cit9] Ruiz de Luzuriaga A., Martin R., Markaide N., Rekondo A., Cabanero G., Rodriguez J., Odriozola I. (2016). Mater. Horiz..

[cit10] Yuan Y., Sun Y., Yan S., Zhao J., Liu S., Zhang M., Zheng X., Jia L. (2017). Nat. Commun..

[cit11] Johnson L. M., Ledet E., Huffman N. D., Swarner S. L., Shepherd S. D., Durham P. G., Rothrock G. D. (2015). Polymer.

[cit12] Takahashi A., Ohishi T., Goseki R., Otsuka H. (2016). Polymer.

[cit13] Maeda T., Otsuka H., Takahara A. (2009). Prog. Polym. Sci..

[cit14] Bowman C. N., Kloxin C. J. (2012). Angew. Chem., Int. Ed..

[cit15] Kuang X., Liu G., Dong X., Wang D. (2017). Mater. Chem. Front..

[cit16] Jin Y., Yu C., Denman R. J., Zhang W. (2013). Chem. Soc. Rev..

[cit17] Kuang X., Liu G. M., Dong X., Liu X. G., Xu J. J., Wang D. J. (2015). J. Polym. Sci., Part A: Polym. Chem..

[cit18] Kuang X., Liu G., Dong X., Wang D. (2016). Macromol. Mater. Eng..

[cit19] Yu K., Taynton P., Zhang W., Dunn M. L., Qi H. J. (2014). RSC Adv..

[cit20] García J. M., Jones G. O., Virwani K., McCloskey B. D., Boday D. J., ter Huurne G. M., Horn H. W., Coady D. J., Bintaleb A. M., Alabdulrahman A. M. S., Alsewailem F., Almegren H. A. A., Hedrick J. L. (2014). Science.

[cit21] Montarnal D., Capelot M., Tournilhac F., Leibler L. (2011). Science.

[cit22] Zhu C., Xi C., Doro W., Wang T., Zhang X., Jin Y., Zhang W. (2017). RSC Adv..

[cit23] Taynton P., Zhu C., Loob S., Shoemaker R., Pritchard J., Jin Y., Zhang W. (2016). Polym. Chem..

[cit24] Röttger M., Domenech T., van der Weegen R., Breuillac A., Nicolaÿ R., Leibler L. (2017). Science.

[cit25] Taynton P., Ni H., Zhu C., Yu K., Loob S., Jin Y., Qi H. J., Zhang W. (2016). Adv. Mater..

[cit26] Taynton P., Yu K., Shoemaker R. K., Jin Y., Qi H. J., Zhang W. (2014). Adv. Mater..

[cit27] Yu K., Shi Q., Dunn M. L., Wang T. J., Qi H. J. (2016). Adv. Funct. Mater..

[cit28] Shi Q., Yu K., Dunn M. L., Wang T., Qi H. J. (2016). Macromolecules.

[cit29] Park G. S. and Crank J., Diffusion in polymers, 1968

[cit30] Tu Y.-O., Ouano A. (1977). IBM J. Res. Dev..

[cit31] Ribar T., Bhargava R., Koenig J. L. (2000). Macromolecules.

[cit32] Lee P. I. (1980). J. Membr. Sci..

[cit33] Peppas N. A., Wu J., von Meerwall E. D. (1994). Macromolecules.

[cit34] May C. , Epoxy resins: chemistry and technology, CRC press, 1987

[cit35] El Gersifi K., Durand G., Tersac G. (2006). Polym. Degrad. Stab..

[cit36] Khawam A., Flanagan D. R. (2006). J. Phys. Chem. B.

[cit37] Freedman B., Butterfield R. O., Pryde E. H. (1986). J. Am. Oil Chem. Soc..

[cit38] Kafka S., Larissegger-Schnell B., Kappe T. (2004). J. Heterocycl. Chem..

[cit39] Pratt R. C., Lohmeijer B. G., Long D. A., Waymouth R. M., Hedrick J. L. (2006). J. Am. Chem. Soc..

[cit40] Hansen C. M. , Hansen solubility parameters: a user's handbook, CRC press, 2007

[cit41] Launay H., Hansen C. M., Almdal K. (2007). Carbon.

[cit42] Rubinstein M., Semenov A. N. (1998). Macromolecules.

[cit43] Stauffer D. (1979). Phys. Rep..

[cit44] Liu T., Zhang M., Guo X., Liu C., Liu T., Xin J., Zhang J. (2017). Polym. Degrad. Stab..

[cit45] Yang H., Yu K., Mu X., Wei Y., Guo Y., Qi H. J. (2016). RSC Adv..

[cit46] Long R., Qi H. J., Dunn M. L. (2013). Soft Matter.

[cit47] Wellek R. M., Mitchell R. D., Moore J. W. (1971). J. Chem. Eng. Data.

