# Supplementary material for: Dissolution of epoxy thermosets via mild alcoholysis: the mechanism and kinetics study
Source: RSC Adv. 2018 Jan 4;8(3):1493–502. doi: 10.1039/c7ra12787a (PMC9077052; doi:10.1039/c7ra12787a)
Supplement: RA-008-C7RA12787A-s001 [file RA-008-C7RA12787A-s001.pdf]

Electronic Supplementary Material (ESI) for RSC Advances.
This journal is © The Royal Society of Chemistry 2017

Supporting Information

Dissolution of Epoxy Thermosets via Mild Alcoholysis: the Mechanism and Kinetics Study

Xiao Kuang[a,b], Qian Shi[a,c], Yunying Zhou[a,d], Zeang Zhao[a,e], Tiejun Wang[c], H. Jerry Qi[a,b]*

[a] The George W. Woodruff School of Mechanical Engineering
[b] Renewable Bioproduct Institute
Georgia Institute of Technology, Atlanta, GA 30332, USA
[c] State Key Lab for Strength and Vibration of Mechanical Structures
School of Aerospace Engineering, Xian Jiaotong University, Xian 710049, China
[d] Department of Architectural Engineering
North China Institute of Aerospace Engineering, Langfang 065000, China
[e] College of Engineering, Peking University, Beijing 100871, P. R. China.
*E-mail: qih@me.gatech.edu

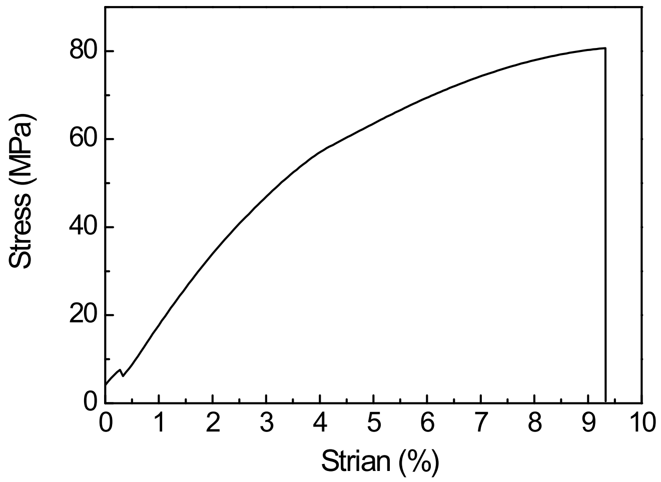

Figure S1 The tensile stress-strain curves of anhydride-epoxy thermosets.

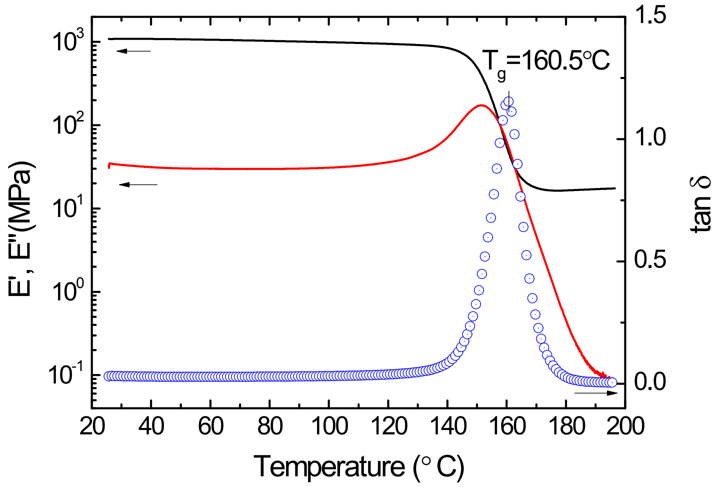

Figure S2 DMA heating curve of anhydride-epoxy thermosets.

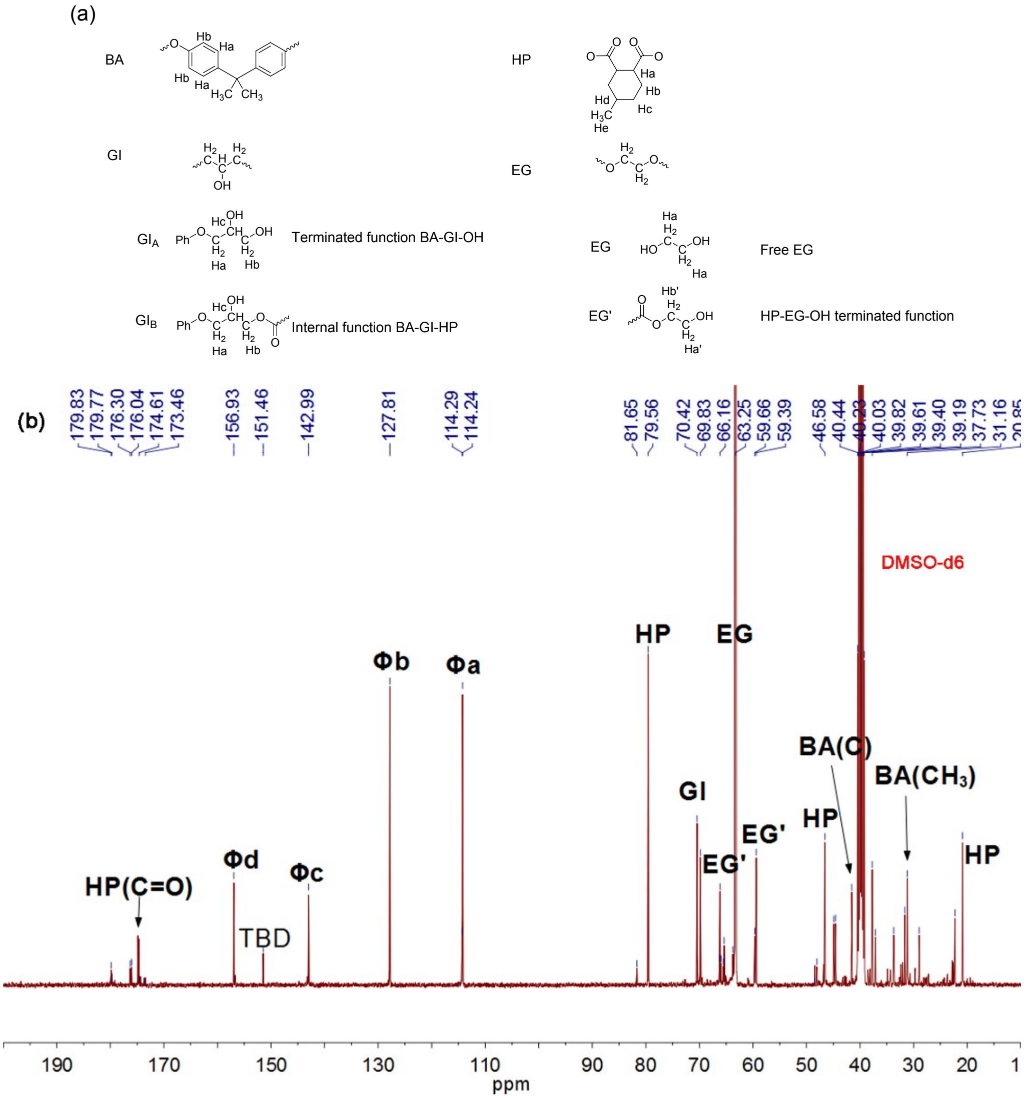

Figure S3 (a) Proposed structures formula of recycled epoxy oligomer and position of protons for NMR study. (b) ^{13}C NMR spectrum of the recycling solution with an epoxy solid content of 15 wt% in 0.35M TBD-EG solution after treating at 170 ^{o}C for 1 h.

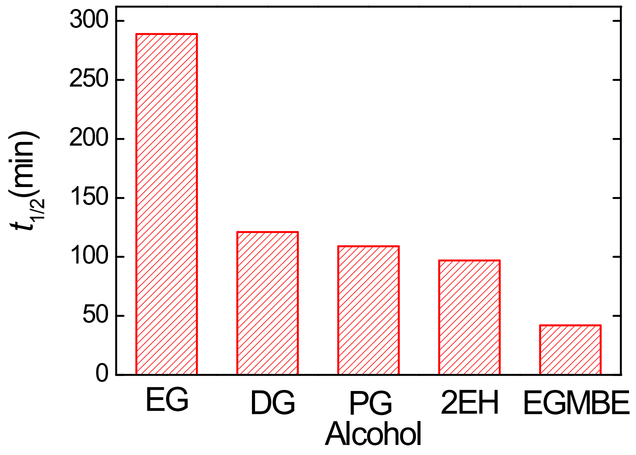

Figure S4 Dissolution half time ($t_{1/2}$) of epoxy in 0.35M TBD-alcohol solutions at 160 °C.

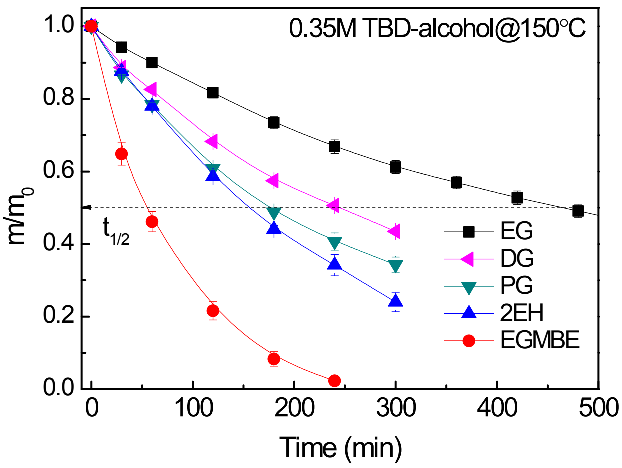

Figure S5 Normalized residual mass of epoxy sample in 0.35M TBD-alcohol solutions at 150 °C with different alcohols as a function of heating time

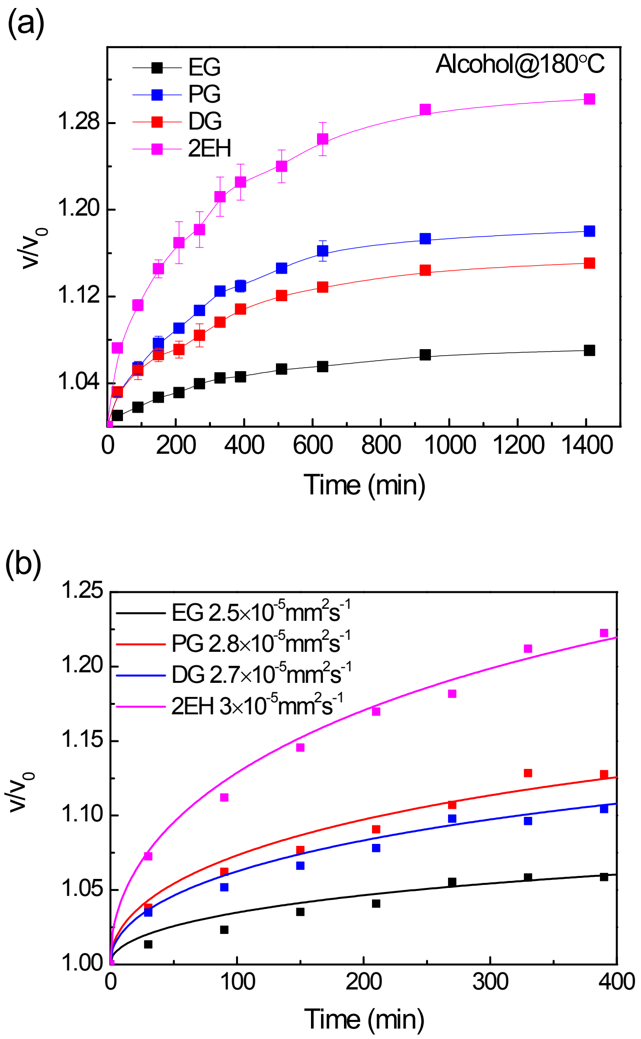

Figure S6 (a) The time-dependent swelling ratio of epoxy resin in different alcohols at 180 °C. (b) The experimental data of swelling within 400 min was fitted by Fick's second law to obtain *D*.

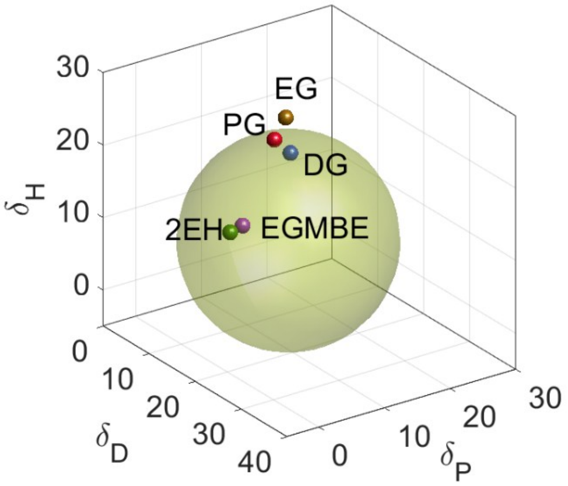

Figure S7 Epoxy resin (solubility sphere) and several alcohols (points) in Hansen space. δ_D, δ_P, and δ_H are the dispersion (nonpolar), permanent-dipole, and hydrogen bonding solubility parameters, respectively.

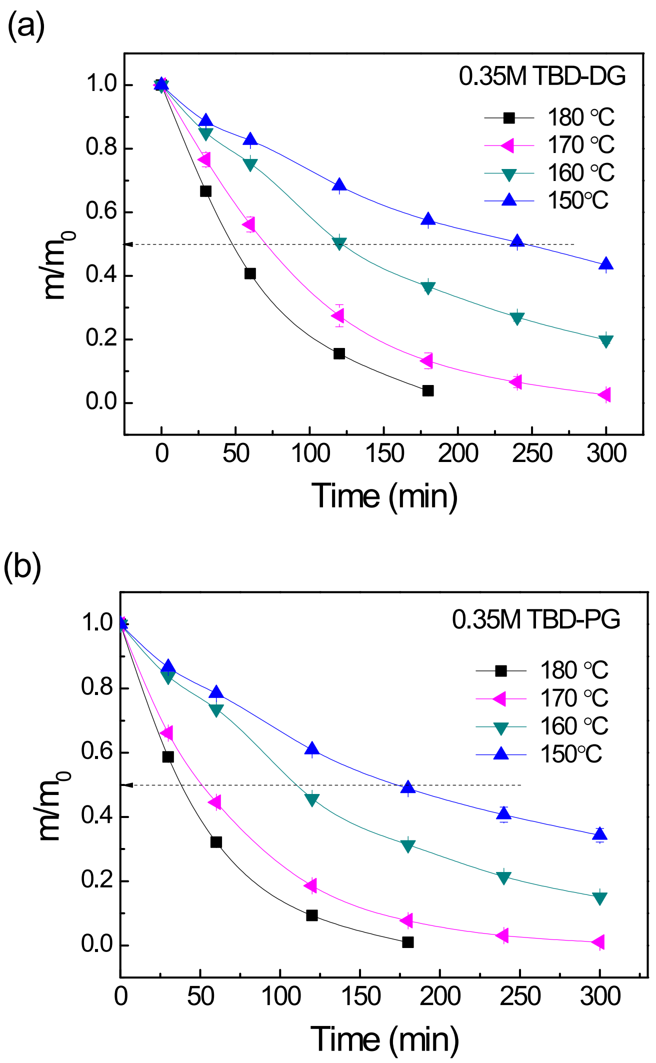

Figure S8 Normalized residual mass of epoxy sample in TBD-alcohol solution at different temperature as a function of heating time: (a) DG, (b) PG.

Hansen solubility parameters (HSP) analysis

The solubility of solvent to epoxy can be evaluated by Hasan solubility parameters (HSP) [1]. The distance parameter between the solvent and the material is calculated according to the follow equation:

$$R_a^2 = 4(\delta_{D1} - \delta_{D2})^2 + (\delta_{P1} - \delta_{P2})^2 + (\delta_{H1} - \delta_{H2})^2 \quad \text{(S1)}$$

where R_a is a modified difference between the HSP for a solvent and polymer, δ_D, δ_P, and δ_H are the contributions from nonpolar (dispersion) forces (D), permanent-dipole forces (P) and hydrogen-bonding (H) effects, respectively.

The ratio between R_a and R_o (the sphere of interaction for the materials) is called the relative energy difference (RED).

$$RED = \frac{R_a}{R_o} \quad \text{(S2)}$$

Good solvents are found within the sphere of affinity which have a RED number less than 1.0. Solvents giving higher RED numbers indicate lower solubility. Based on the Flory–Huggins model, the relationship between the Flory–Huggins interaction parameter (χ) and the HSP solubility parameter is described by the following equation[2]:

$$\chi = \beta V_1 \frac{R_a^2}{4} / RT \quad \text{(S3)}$$

where β is a constant value (=0.6), V_1 is the molar volume of solvent, R is the gas constant, and T is the absolute temperature.

Dissolution kinetics based on surface layer model

For solid-state kinetic analysis, the mass loss or the conversion fraction (α) can be defined as:

$$\alpha = \frac{m_0 - m_t}{m_0} = 1 - \frac{m_t}{m_0} \quad \text{(S4)}$$

where m_0 is the initial weight, m_t is the weight at time t.

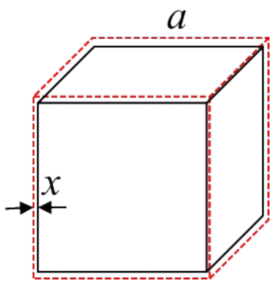

Scheme S1. The side length of a cubic sample (with an original size of *a*) changes *x*.

For a cubic sample (side length of *a*) with the reaction depth of *x*, α is expressed as：

$$\alpha = \frac{a^3 - (a-x)^3}{a^3} \quad \text{(S5)}$$

So we can get

$$a - x = a(1-\alpha)^{1/3} \quad \text{(S6)}$$

The new surface with 6 square faces has the total area of

$$A = 6(a-x)^2 = 6a^2(1-\alpha)^{2/3} \quad \text{(S7)}$$

Based on the general solid-state reaction kinetics described by an order-based model, we obtain the following kinetics equation[3]:

$$\frac{d\alpha}{dt} = k_n A(1-\alpha)^n = 6a^2 k_n (1-\alpha)^{2/3+n} \quad \text{(S8)}$$

where k_n is the rate constant of reaction order n.

If the dissolution rate is controlled by the resulting reaction interface toward the center of the sample, n is 0. Eq. S8 is simplified as:

$$\frac{d\alpha}{dt} = 6a^2 k (1-\alpha)^{2/3} \quad \text{(S9)}$$

The integration of Eq. S9 gives the following equation:

$$1-(1-\alpha)^{1/3} = 2ka^2 t \quad \text{(S10)}$$

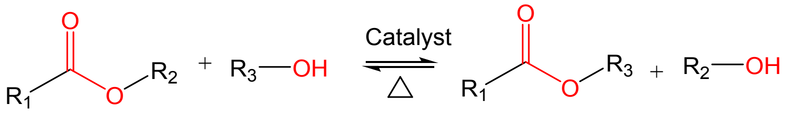

Scheme S2. The transesterification reaction between ester and alcohol catalyzed by catalyst at elevated temperature.

Based on the reaction kinetics, the transesterification rate is proportional to the hydroxyl

concentration, ester concentration and ER rate constant (k_{ER}). If alcohol is in excess, the reverse reaction can be ignored and the total reaction rate can be expressed:

$$-\frac{d[C=O]}{dt} = k_{ER}[C=O][OH] \tag{S11}$$

where [C=O] is the concentration of ester group, [OH] is the concentration of hydroxyl group, and k_{ER} is the ER rate constant.

For the proposed surface layer model with a thin reactive layer (δ), we can express the solid-state reaction kinetics in a homogeneous transesterification reaction kinetics:

$$\frac{d\alpha}{dt} \sim \frac{d[C=O]}{dt}V_{surf} = k_{ER}[C=O][OH]V_{surf} \sim \frac{\varphi_1(1-\varphi_1)\eta}{V_1V_2}k_{ER}V_{surf} \tag{S12}$$

$$V_{surf} = A\delta = 6a^2\delta(1-\alpha)^{2/3} \tag{S13}$$

where A is the surface area at time t, η is functionality of hydroxyl group in alcohol, φ_1 is the solvent fraction in the swallowing layer after equilibrium, V_2 is molar volume of polymer ((1-φ_1)/V_2: the ester concentration of swollen polymer, V_1 is molar volume of alcohol (φ_1/ V_1: the hydroxyl group concentration in the swollen polymer), V_{surf} is the total surface volume within the reactive layer of δ.

Based on Fick diffusion law, the diffusion distance is proportional to the square-root of diffusion coefficient D[4]:

$$\delta \sim D^{1/2} \tag{S14}$$

We can obtain the dissolving rate by the following equation:

$$\frac{d\alpha}{dt} \sim \frac{\varphi_1(1-\varphi_1)a^2}{V_1V_2}k_{ER}D^{1/2}(1-\alpha)^{2/3} \tag{S15}$$

Generally, the temperature dependent transesterification kinetics can be described using Arrhenius equation:

$$k_{ER} = k_{ER,0}\exp(\text{-}\Delta E_{ER}/RT) = \varphi_1 k_{ER}{}' = \varphi_1 k_{ER,0}{}'\exp(\text{-}\Delta E_{ER}/RT) \tag{S16}$$

The mutual-diffusion coefficient (D) of epoxy-solvent can be expressed in the following equation[5-6]:

$$D = D_1(1-\phi_1)^2(1-2\chi\phi_1) \tag{S17}$$

$$D_1 = D_0\exp(\text{-}\frac{\Delta E_{\mathrm{d}iff}}{RT}) \tag{S18}$$

where D_1 is the solvent self-diffusion coefficient, φ_1 is the solvent volume fraction and χ is Flory-Huggins solubility parameter, ΔE_{ER} is the ER activation energy, ΔE_{diff} is the diffusion activation energy.

Substituting Equation S16 and S18 into Equation S15, and solve the differential equation, we have:

$$1-\alpha = \left[1 - C\frac{\varphi_1{}^2(1-\varphi_1)a^2k_{ER,0}{}'D_0{}^{1/2}}{V_1V_2}\exp\left(\text{-}\left(\Delta E_{ER} + \frac{1}{2}\Delta E_{\mathrm{d}iff}\right)/RT\right)t\right]^3 \tag{S18}$$

Or

$$1-(1-\alpha)^{1/3} = C_0\exp\left(-\frac{\Delta E}{RT}\right)t \tag{S19}$$

With $\Delta E = \Delta E_{ER} + \frac{1}{2}\Delta E_{diff}$, $C_0 = \frac{\varphi_1^{\ 2}(1-\varphi_1)a^2 k_{ER,0}^{'} D_0^{\ 1/2}}{V_1 V_2}$.

Reference

(1) Hansen, C. M., *Hansen solubility parameters: a user's handbook*. CRC press: 2007.

(2) Lindvig, T.; Michelsen, M. L.; Kontogeorgis, G. M. A Flory–Huggins model based on the Hansen solubility parameters. *Fluid Phase Equilibria* **2002,** *203*(1), 247-260.

(3) Khawam, A.; Flanagan, D. R. Solid-state kinetic models: basics and mathematical fundamentals. *J. Phys. Chem. B* **2006,** *110*(35), 17315-17328.

(4) Crank, J., *The mathematics of diffusion*. Oxford university press: 1979.

(5) Duda, J.; Vrentas, J.; Ju, S.; Liu, H. Prediction of diffusion coefficients for polymer - solvent systems. *AIChE Journal* **1982,** *28*(2), 279-285.

(6) Zielinski, J. M.; Duda, J. L. Predicting polymer/solvent diffusion coefficients using free-volume theory. *AIChE Journal* **1992,** *38*(3), 405-415.
